# Identification of a conserved drug binding pocket in TMEM16 proteins

**DOI:** 10.21203/rs.3.rs-1296933/v1

**Published:** 2022-02-10

**Authors:** Yifan Cheng, Shengjie Feng, Cristina Puchades, Juyeon Ko, Eric Figueroa, Yifei Chen, Hao Wu, Shuo Gu, Tina Han, Junrui Li, Brandon Ho, Brian Shoichet, Yuh Nung Jan, Lily Jan

**Affiliations:** University of California San Francisco; UCSF; UCSF; UCSF; UCSF; UCSF; University of California San Francisco; UCSF; UCSF; UCSF; UCSF; UCSF; UCSF; University of California, San Francisco

## Abstract

The TMEM16 family of calcium-activated membrane proteins includes ten mammalian paralogs (TMEM16A-K) playing distinct physiological roles with some implicated in cancer and airway diseases. Their modulators with therapeutic potential include 1PBC, a potent inhibitor with anti-tumoral properties, and the FDA-approved drug niclosamide that targets TMEM16F to inhibit syncytia formation induced by SARS-CoV-2 infection. Here, we report cryo-EM structures of TMEM16F associated with 1PBC and niclosamide, revealing that both molecules bind the same drug binding pocket. We functionally and computationally validate this binding pocket in TMEM16A as well as TMEM16F, thereby showing that drug modulation also involves residues that are not conserved between TMEM16A and TMEM16F. This study establishes a much-needed structural framework for the development of more potent and more specific drug molecules targeting TMEM16 proteins.

TMEM16 proteins are a family of transmembrane proteins conserved across eukaryotes, encompassing 10 paralogs (TMEM16A-K) in mammals. Despite high sequence similarity, TMEM16 proteins present remarkable functional diversity (1, 2). For instance, TMEM16A is a calcium (Ca^2+^)-activated chloride channel that opens in response to increased intracellular Ca^2+^ levels, enabling chloride ions to move across the plasma membrane (3–5). In contrast, TMEM16F functions as both a Ca^2+^-activated ion channel and a Ca^2+^-activated lipid scramblase. TMEM16F channels permeate cations including Ca^2+^ ions (6–8), however, its selectivity for cations versus anions may vary with the electrostatic field of the permeant pathway (9). Through its lipid scrambling activity, TMEM16F allows diverse lipids, including phosphatidylcholine (PC), phosphatidylethanolamine (PE) and phosphatidylserine (PS), to passively move between the inner and outer leaflets of the plasma membrane (1, 2, 6, 7, 10, 11). Both TMEM16A and TMEM16F play critical roles in numerous physiological processes and have emerged as important targets for therapeutic intervention in multiple diseases, including cancer, asthma, and in particular COVID-19 (1, 2, 12, 13).

TMEM16A is required for airway and secretory gland secretion (1, 2). Notably, a TMEM16A activator is under consideration for the treatment of cystic fibrosis (14), whereas TMEM16A inhibitors with potent bronchodilator activities are being tested as anti-asthma drugs (15, 16). Additionally, TMEM16A activity is upregulated via gene amplification or enhanced expression in many types of cancers and is linked to increased cell migration and proliferation as well as metastatic progression (12). Therefore, antagonists of TMEM16A, such as 1PBC and niclosamide, provide a promising new avenue for the treatment of diverse cancers (16).

TMEM16F activity is important for blood coagulation (6, 17–19) and mutations in TMEM16F are linked to the Scott syndrome bleeding disorder (20). TMEM16F also plays a critical role in extracellular vesicle generation and release (21 –24) and membrane repair as protection against bacterial infection (25). Importantly, niclosamide, an FDA approved drug, has recently been shown to block SARS-CoV-2-induced syncytia formation and virus replication by inhibiting TMEM16F (13). The repurposing of niclosamide for treatment of severe COVID-19 is currently under examination in more than a dozen clinical trials (26) (clinicaltrials.gov). Niclosamide is also a potent inhibitor of TMEM16A and robustly mitigates the symptoms of airway diseases in mice (15). Like niclosamide, most small molecule inhibitors identified to date affect multiple TMEM16 paralogs, making off-target effects a major concern for clinical applications. Elucidation of the ligand binding site is important not only for understanding the mechanism of action of these molecules but also for designing more specific drugs for pharmacological targeting of TMEM16 proteins.

TMEM16 proteins form dimers (1, 2); each subunit comprises 10 transmembrane helices (TMs) and contains its own ion conduction pore enclosed and surrounded by TM3–7 (Fig. 1). Ca^2+^-dependent activation involves direct binding of Ca^2+^ ions in two contiguous Ca^2+^-binding sites that are formed between TM6-TM8 (Fig. 1). Structures of both TMEM16A and TMEM16F in Ca^2+^-free and Ca^2+^-bound states reveal that TM6 undergoes major Ca^2+^-dependent conformational rearrangements, whereby Ca^2+^ binding stabilizes an extended conformation of TM6 (8, 27, 28). While numerous studies have established a critical role for TM6 in binding Ca^2+^ for channel activation (1, 2), it is an intriguing open question as to how TMEM16A and TMEM16F functions might be modulated by small molecule drugs.

We determined cryo-EM structures of TMEM16F in three distinct unliganded states that reveal structural asymmetry and shed light into the mechanisms underlying Ca^2+^-activated lipid scrambling. We also determined structures of TMEM16F bound to niclosamide and 1PBC, revealing that both molecules bind the same hydrophobic groove. We validated the binding site using computational docking and mutagenesis analyses and our data also indicate that both niclosamide and 1PBC bind to the equivalent site in TMEM16A. Our work establishes a structural foundation for designing more potent and specific antagonists against TMEM16 proteins with critical implications for the treatment of cancer, asthma and COVID-19.

## Results

### Cryo-EM analysis reveals an asymmetric state of the TMEM16F dimer

A plethora of genetic, biochemical and electrophysiological studies show that binding of phosphatidylinositol 4,5-biphosphate (PIP_2_) is important for activation of both TMEM16A and TMEM16F (29–31). Combination of lipid nanodisc technology with single particle cryo-EM allows structural analysis of membrane proteins embedded in a lipid bilayer (32, 33), which is critical for TMEM16 proteins and other membrane proteins that are modulated by lipids. However, TMEM16 proteins in nanodiscs present strong preferred orientation in particle distribution, severely limiting the attainable resolution of cryo-EM structures of TMEM16 proteins and hampering the study of these proteins in the context of a lipid bilayer (8, 27). We overcame this limitation by collecting data from tilted specimen and implementing an image processing pipeline that allowed us to systematically determine sub 3.5 Å structures of TMEM16F in lipid nanodiscs in the presence or absence of different ligands (see Materials and Methods, figs. S1, S2, S3, S4 and table S1).

**Figure 1:**
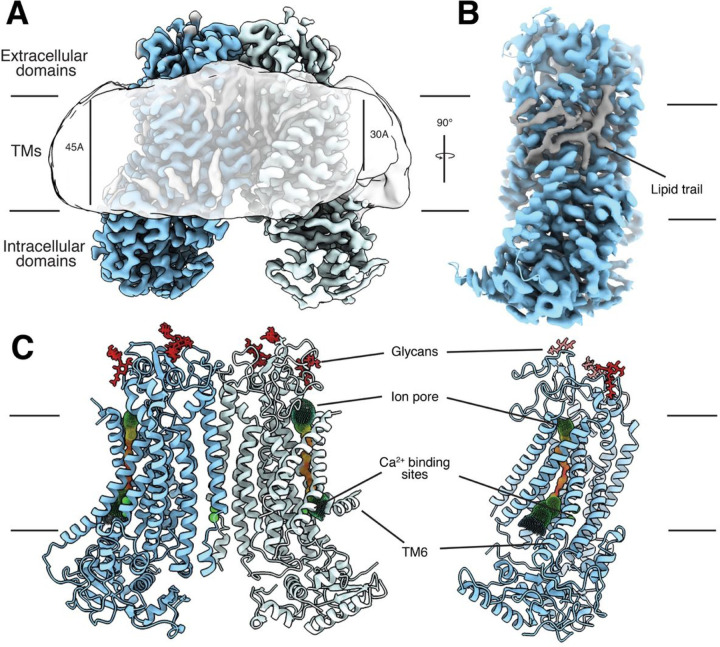
Cryo-EM analysis reveals asymmetry within the TMEM16F dimer. (**A**) Cryo-EM density of the asymmetric state of the TMEM16F dimer with the monomers colored blue and light blue, respectively, and the lipid densities in grey. The gaussian filtered cryo-EM density (semitransparent) reveals distortion of the lipid nanodisc. (**B**) Side view of a TMEM16F monomer (blue) highlighting the trail of lipids (grey) covering the TM region. (**C**) Front and side view of the atomic model of the asymmetric state of TMEM16F with Ca^2+^ atoms and glycans shown in green and red, respectively. The ion conduction channel identified by HOLE is represented by spheres colored in rainbow scale based on the local width of the channel, where red <1.5 Å and blue >7.5 Å.

First, we determined multiple structures of TMEM16F in the presence of Ca^2+^ and PIP_2_. These structures represent different conformations of TMEM16F in unliganded states. The quality of these reconstructions enables atomic model building of the TM helices, most of the extracellular and intracellular domains, as well as Ca^2+^ ions and dozens of lipid densities associated with the protein (Fig. 1 and table S1). Whereas all previously reported TMEM16F structures were determined with the assumption of C2 symmetry, we did not impose symmetry and identified 3 distinct states with major differences in the conformation of TM6 and the number of Ca^2+^ atoms bound in each monomer (fig. S4). In State A, both monomers are bound to 2 Ca^2+^ ions and present a clear density for an extended TM6 (fig. S4). In State B, one monomer has 2 Ca^2+^ ions and a straight TM6, whereas the other monomer appears to contain a single Ca^2+^ ion, as density for the second Ca^2+^ ion is significantly weaker. In this single Ca^2+^-bound monomer, TM6 presents a kink at P628 (Fig. 1). Thus, this structure represents an asymmetric state of the dimer (Fig. 1). In State C, both monomers contain only 1 Ca^2+^ ion and TM6 is bent in both (fig. S4). Comparison between these 3 classes reveals that straightening of TM6 correlates with binding of the second Ca^2+^ ion, whereas kinking of TM6 is associated with an outward rigid body motion of the intracellular domain that brings it closer to the nanodisc (Fig. 1A and fig. S4). Moreover, bending of TM6 directly correlates with distortion of the nanodisc and significant thinning of the membrane at the kinking position (Fig. 1A and fig. S4). Consistent with our previous study of TMEM16F (8), these observations support the notion that kinking of TM6 at P628 causes membrane distortion.

Our reconstructions also reveal previously unobserved features, including glycans and conserved disulfide bonds in the extracellular region (Fig. 1 and fig. S5, A and B), as well as the presence of a third Ca^2+^ ion coordinated by E395 on TM2 as well as S854 and D859 on TM10, near the dimer interface in the intracellular region of the protein (fig. S5C). These features are likely present in previous reconstructions but not detected due to limited resolution. In fact, a similar Ca^2+^-binding site has been recently found in TMEM16F (10) as well as TMEM16K (34), and biochemical studies indicate that an equivalent third Ca^2+^-binding site allosterically regulates channel activity in TMEM16A (35).

We are also able to unambiguously assign the residues of TM4 and precisely determine the pore-lining residues on TM4 (fig. S5D). These residues form a network of OH-containing side chains along the hydrophilic pore that constitutes an ideal environment for ion conduction across the membrane (Fig. 1C). However, the ion conduction pore is closed in all states resolved in this study and its hydrophilic interior is not accessible to lipids from the surrounding membrane (fig. S5E).

### TM1 and TM6 form a hydrophobic groove that can be occupied by lipids

**Figure 2:**
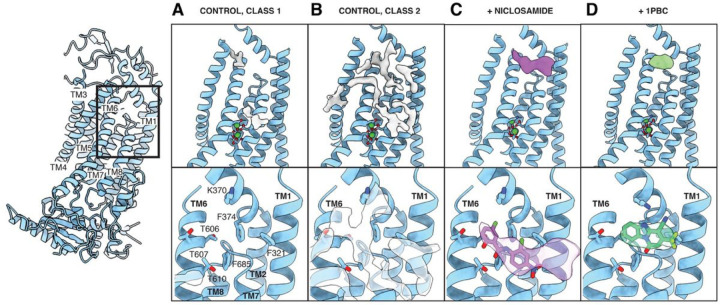
Niclosamide and 1PBC bind the same hydrophobic groove in TMEM16F. Atomic model of the TM1-TM6 region of (**A**) Class1 and (**B**) Class 2 of the drug-free control, (**C**) niclosamide- and (**D**) 1PBC-supplemented datasets. In each case, the additional cryo-EM densities found in the area are shown. Below, zoom into the TM1-TM6 groove with the residues shown as sticks and colored by heteroatom and the additional density found within the pocket shown in semitransparent. Structures of niclosamide and 1PBC as determined by computational docking using Glide are shown in purple and green, respectively.

In all three classes, we noticed a trail of densities that appear to correspond to a mixture of multiple lipids extending across the entire lipid bilayer along the membrane-facing surface of each TMEM16F monomer (Fig. 1B). A hydrophobic groove formed between TM1 and TM6 near the extracellular edge of the membrane appears to play a major role in accommodating these lipids. Intriguingly, this area corresponds to the position where membrane thinning occurs. To further investigate these lipid densities, we combined particles from all three States and carried out focused classification around this groove in a single monomer (See Materials and Methods, fig. S1). The particles clustered primarily to 2 classes that each contained approximately 40% of the particles and rendered 3.1 Å resolution structures (figs. S1 and S2). The overall organization of Class 1 and 2 is essentially indistinguishable (Fig. 2). However, Class 1 almost entirely lacks lipid densities in the TM1-TM6 groove, whereas Class 2 has strong density for numerous lipids in this area (Fig. 2, A and B). This indicates that our dataset contains a mixture of monomers in lipid-free and lipid-bound states.

### Niclosamide binds the hydrophobic groove formed between TM1 and TM6

Niclosamide is an FDA-approved drug that has recently emerged as a promising drug for treating severe cases of COVID-19 (26) (clinicaltrials.gov), and its propensity to inhibit syncytia formation has been attributed to its ability to inhibit TMEM16F (13). Seeking to determine the binding site of this antagonist, we added 50 mM niclosamide to our biochemical preparation and imaged this sample following identical image processing pipeline as in the apo dataset presented above (figs. S1 and S2). In this case, however, focused classification around the TM1-TM6 groove rendered 3 classes. Like in our control sample, Classes 1 and 2 are distinguished by the absence or presence of lipids in the groove. Meanwhile, Class 3 contains a well-defined density in the TM1-TM6 groove that fits niclosamide well while no trail of lipid densities is found in the hydrophobic pocket (Fig. 2C). The niclosamide-like density contacts F321 on TM1, K370 on the TM1-TM2 loop, T606, T607 and T610 on TM6, and F685 and L687 on the TM7-TM8 loop (Fig. 2C). The resolution of our reconstruction is insufficient to unambiguously determine the precise pose of the molecule within the density. To gain some insight into how niclosamide may be oriented within TMEM16F, the compound was computationally docked using the Glide docking software. Using only the atomic model of TMEM16F (without access to our cryo-EM density map), the software identified this pocket as the most likely binding site and the highest-ranking pose fits our cryo-EM density well (Fig. 2C and fig. S6). Notably, this pose had the lowest binding energy and predicts formation of a hydrogen bond with T610. Taken together, our structural and computational data show that niclosamide binds TMEM16F at the hydrophobic groove formed between TM1 and TM6 and that binding of niclosamide prevents lipids from occupying this pocket.

### 1PBC is a potent inhibitor of TMEM16F

**Figure 3:**
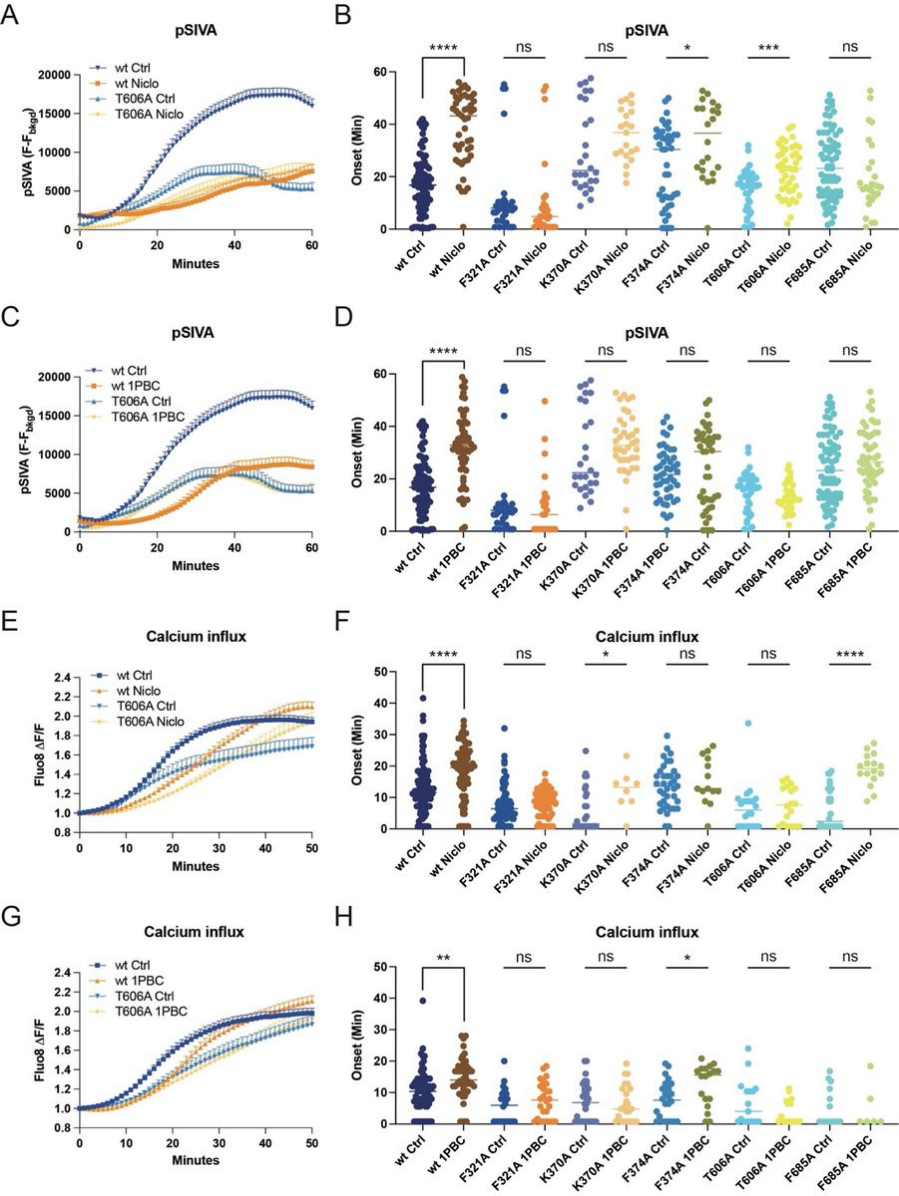
Functional validation of the drug binding site in TMEM16F. Representative curves of live imaging of TMEM16F-dependent PS exposure (**A**) and (**C**); and Ca^2+^ influx (**E**) and (**G**). Data are represented as mean ± SEM. Scattered dot plots of time of onset of TMEM16F-dependent PS exposure [(**B**) and (**D**)] and Ca^2+^ influx [(**F**) and (**H**)]. Time of onset could not be determined for time courses with a linear rather than sigmoidal rise. The mean ± SEM is shown along with the statistical significance determined by unpaired t-test for each mutant as compared to vehicle controls (*p < 0.05; **p < 0.01; ***p < 0.001; ****p < 0.0001).

Niclosamide is known to inhibit both TMEM16F and TMEM16A channels (15). Given the structural similarities between both paralogs, we reasoned that 1PBC, a potent inhibitor of TMEM16A, might also modulate TMEM16F. To test this hypothesis, we first measured Ca^2+^ influx using Fluo8 as a small molecule Ca^2+^ reporter dye. Application of 1PBC led to a significant decrease in TMEM16F-dependent Ca^2+^ influx upon chemical induction (Fig. 3 and fig. S7). This indicates that 1PBC is a potent inhibitor of TMEM16F ion channel activity. Next, we explored whether TMEM16F lipid scramblase activity is also inhibited by 1PBC by imaging PS exposure using pSIVA, a fluorescent annexin derivative. Upon chemical induction, the average onset for PS exposure in vehicle controls was 17.23 min (Fig. 3 and fig. S7). 1PBC robustly delayed the onset of TMEM16F-dependent PS exposure to 32.06 min (Fig. 3). We conclude that, like niclosamide, 1PBC potently inhibits TMEM16F function by reducing both ion conduction and lipid scrambling activity.

### 1 PBC and niclosamide target the same site in TMEM16F

To elucidate the binding site of 1PBC, we supplemented our TMEM16F sample with 100 mM 1PBC. Here too we identified 3 distinct classes that closely resemble the 3 states observed in our drug-free sample. However, lipid densities along the membrane-facing surface of each monomer are absent. Instead, in all three classes we found a strong oval-shaped density in the same hydrophobic groove identified as the drug binding site in our niclosamide-supplemented dataset (Fig. 2D). This density, which is remarkably different from the lipid-like and niclosamide-like densities in our ligand-free and niclosamide-bound structures, fits 1PBC well. Overlay of the 1PBC-bound structure with our control revealed subtle side chain rearrangements of the residues surrounding this density. More specfically, K370 appears to shift from interacting with E366 to establishing a hydrogen bond with the compound (fig. S5F). Consistent with these observations, computational docking using Glide independently predicts formation of a hydrogen bond between K370 and 1PBC and identifies a pose for the ligand that fits our density map well (Fig. 2D and fig. S6). Together, our data show that 1PBC and niclosamide target the same site in TMEM16F and appear to replace bound lipids in the hydrophobic groove formed between TM1 and TM6.

### Functional validation of the drug binding site in TMEM16F

We previously showed that chemical induction of giant plasma membrane vesicle formation involves TMEM16F-dependent Ca^2+^ influx as well as TMEM16F-dependent PS exposure in HEK293 cells (7, 8), so it is a robust assay for evaluating TMEM16F activity. We thus generated stable cell lines expressing wildtype or mutant TMEM16F-mScarlet containing alanine substitutions of the residues surrounding the inhibitor densities: F321 on TM1, K370 and F374 on the TM1-TM2 loop, T606 on TM6, and F685 on the TM7-TM8 loop. Interestingly, mutation of these residues altered the basal activity of TMEM16F. Compared to the wildtype control, F321A shortened the onset of Ca^2+^ influx by nearly twofold and reduced the onset latency of the PS exposure from 17.23 min to 11.61 min (Fig. 3). In contrast, K370A significantly delayed the onset of PS exposure to 30.16 min. These results indicate that this pocket and its endogenous lipids are critical for scramblase activity of TMEM16F (Fig. 3 and fig. S7). Importantly, in wild type controls, both 1PBC and niclosamide significantly delayed the onset of internal Ca^2+^ rise and PS exposure (Fig. 3). The inhibitory effect of both antagonists was significantly decreased by all the mutations, confirming that these residues are important for binding these inhibitors (Fig. 3). In fact, the F321A mutation almost completely obliterated the inhibitory effect on the onset of both Ca^2+^ rise and PS exposure (Fig. 3). In summary, we show that residues in the TM1-TM6 groove are important for niclosamide- and 1PBC-mediated inhibition of TMEM16F and this area is critical for scramblase activity.

### Functional and computational validation of the niclosamide and 1PBC binding site in TMEM16A

**Figure 4:**
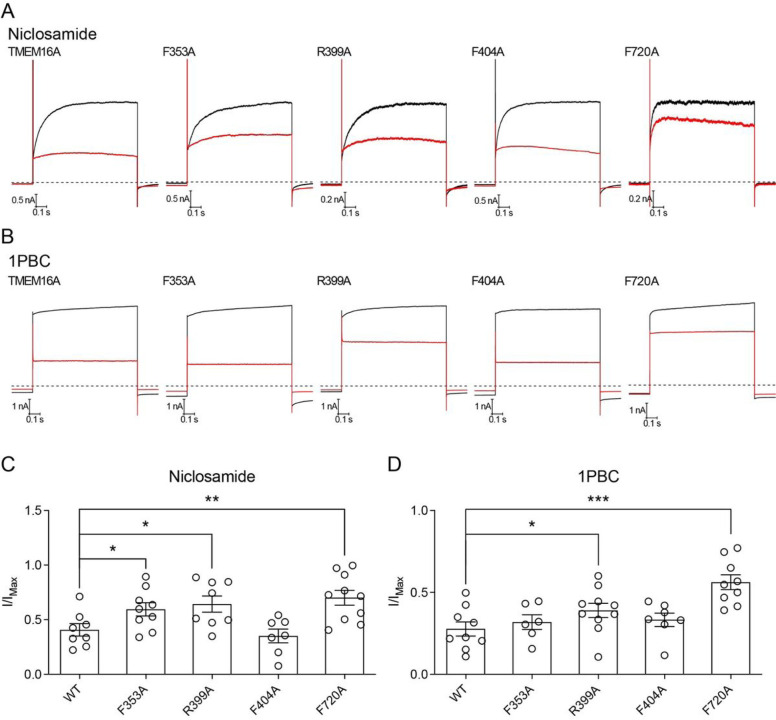
Electrophysiology-based validation of the binding site in TMEM16A. Representative voltage clamp current traces at +70 mV with holding potential at −5 mV of wildtype mTMEM16A and alanine substitution mutants in the absence or presence of (**A**) 3 μM niclosamide or (**B**) 30 μM 1PBC recorded in 500 nM [Ca^2+^]_i_ or 12 mM [Ca^2+^]_i_, respectively. Graphs showing the coefficient of the steady-state current measured for the wildtype control and each mutant upon addition of (**C**) niclosamide and (**D**) 1PBC divided by the maximum Intensity (I/I_max_) in each case. The mean ± SEM is shown along with the statistical significance determined by unpaired t-test for each mutant as compared to wildtype controls (*p < 0.05; **p < 0.01; ***p < 0.001).

Niclosamide and 1PBC are potent inhibitors of both TMEM16A and TMEM16F (15). Since the binding pocket we identify in TMEM16F presents a high degree of conservation in TMEM16A, we reasoned that both inhibitors may bind equivalent sites in TMEM16A and TMEM16F. To investigate this hypothesis, we tested whether mutations of residues in the putative binding pocket affect 1PBC- or niclosamide-mediated inhibition of TMEM16A (Fig. 4). We used whole cell patch clamp electrophysiology to measure Ca^2+^-activated Cl^−^ currents from HEK293 cells expressing either wildtype or mutant TMEM16A and tested the effects of alanine substitutions of F353 on TM1, R399 and F404 on the TM1-TM2 loop or F720 on the TM7-TM8 loop. In the absence of antagonists, these mutations did not alter the Cl^−^ current induced by Ca^2+^ activation of TMEM16A. R399A and F720A significantly reduced the inhibitory effects of both 1PBC and niclosamide while F353A affected the inhibitory effects of niclosamide but not 1PBC, confirming that these residues are important for the interaction of these drugs with TMEM16A (Fig. 4, A to D). Notably, F404A did not alter the efficiency of either of the inhibitors, whereas the equivalent mutation in TMEM16F, F374A, decreased inhibition (Fig. 3). It thus appears that the functional relevance of the specific residues within the binding site might vary between TMEM16A and TMEM16F.

We further used Glide to computationally dock niclosamide and 1PBC into the Ca^2+^-bound TMEM16A structure following identical procedures as in TMEM16F, for docking into a cube of 30 Å length on each side. In both cases, the software found binding in this pocket to be most energetically favorable (fig. S6). Taken together, our data indicate that 1PBC and niclosamide bind the same binding pocket in a hydrophobic groove formed between TM1 and TM6 in both TMEM16A and TMEM16F.

## Discussion

TMEM16 proteins assemble as dimers and whether the two monomers function independently or cooperatively is unclear. Unlike all previously solved structures of TMEM16 proteins, our reconstructions of TMEM16F in the presence of PIP_2_ with or without drug molecules reveal a high degree of asymmetry within the dimer (Fig. 1). It is important to note that previous studies of TMEM16 proteins in lipid nanodiscs imposed C2 symmetry during cryo-EM data processing. The asymmetry of TMEM16F dimers we observe in our C1 reconstructions is likely linked to the mechanism(s) underlying TMEM16F function and PIP_2_-mediated activation.

**Figure 5:**
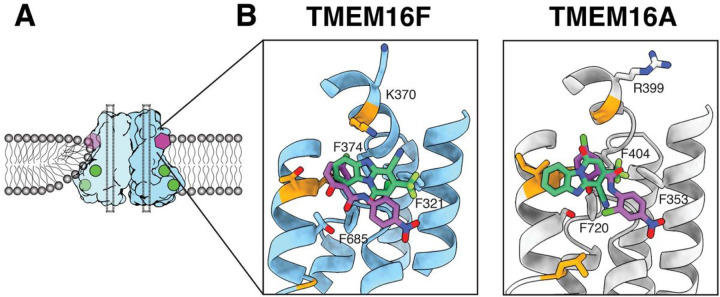
Comparison of the drug binding pocket in TMEM16A and TMEM16F. (**A**) Schematic representation of the TMEM16F dimer (light blue and blue) embedded in a lipid bilayer (grey), where Ca^2+^ atoms are shown as green circles and the inhibitors as a purple polygon and dotted black lines represent the closed ion conduction pore. (**B**) Structure of the drug binding pocket in TMEM16F (blue, left) and TMEM16A (grey, right) with the side chains of the surrounding residues shown as sticks and the non-conserved residues highlighted in orange. Computationally docked structures of niclosamide and 1PBC are shown in purple and green, respectively. All atoms are colored by heteroatom.

Structural analysis of TMEM16F in lipid nanodiscs supplemented with PIP_2_ reveals 3 distinct coexisting states and a direct correlation between kinking of TM6 and membrane distortion (fig. S4). A continuous trail of lipids connects the intra- and extracellular sides of TMEM16F at the membrane distortion site (Fig. 1B). These findings are consistent with our previous structural and mutagenesis data (8) and support a model for TMEM16F-mediated scrambling of lipids, whereby TMEM16F distorts the membrane, minimizing the distance between the inner and outer leaflets of the lipid bilayer (Fig. 5A). We further find that residues along this lipid trail, such as K370 and F321, are important for scramblase activity. In fact, K370 is a positively charged residue that is ideally positioned for interacting with negatively charged phospholipid heads at the membrane interface. The fact that TMEM16A, which cannot scramble lipids, contains an alanine in this position reinforces the notion that this basic residue is critical for lipid scrambling (Fig. 5B). Together, our data suggest that the lipid trail we identify on TMEM16F might correspond to the pathway for lipid scrambling. We propose that the lipids “surf” along this membrane-facing groove, crossing between the inner and outer leaflets through a path that does not directly involve the hydrophilic ion conduction pore (Fig. 5A).

TMEM16 proteins have emerged as important pharmacological targets for the treatment of cancer, asthma and more recently COVID-19 (12, 13) (26) (clinicaltrials.gov). Our data indicate that niclosamide and 1PBC bind the same, conserved site in both TMEM16A and TMEM16F (Fig. 5B and fig. S6). With both antagonists directly contacting the extracellular-proximal end of TM6, our mutagenesis studies suggest that residues on this helix are critical for drug binding (Figs. 3 and 4). TM6 is the main gating element of the channel as well as part of the ion conduction pore in both TMEM16A and TMEM16F (1, 2). We thus speculate that, by binding to the upper part of TM6, these antagonists simultaneously lock the ion conduction pore and the gating element in a closed configuration.

Interestingly, the binding site of 1PBC and niclosamide coincides with the position where we observe the maximum degree of membrane distortion and thinning in the TMEM16F structures. In our model for TMEM16F activity, this site corresponds precisely with the entry and exit point of the lipids as they transition between the inner and outer leaflets of the plasma membrane (Fig. 5A). Our structures show that both antagonists replace the lipids found in this pocket in our drug-free sample (Fig. 2). This suggests that 1PBC and niclosamide might directly inhibit TMEM16F scramblase activity by physically occluding the path of the lipids across the membrane (Fig. 5A). In fact, lipid densities along this path are significantly reduced in our 1PBC- and niclosamide-bound structures (Fig. 2). Consistent with a critical role of this region for lipid scrambling, alanine substitutions of residues within the drug binding pocket significantly alter the lipid scrambling activity of TMEM16F in the absence of inhibitors, whereas equivalent mutations in TMEM16A do not affect Ca^2+^-activated Cl^−^ channel activity (Figs. 3 and 4).

Like 1PBC and niclosamide, most drug molecules identified to date are not specific for any particular TMEM16 paralog and instead broadly target TMEM16 family members. This raises concerns about potential off target effects in the clinic. Additionally, our whole cell patch clamp electrophysiology experiments, as well as previous studies (36), show that niclosamide may activate an ion channel. Although both niclosamide and 1PBC appear to bind the same, conserved hydrophobic pocket in TMEM16A and TMEM16F, our data also show that the specific contribution of different residues to the interaction is distinct in TMEM16A and TMEM16F (Figs. 3 and 4). We further demonstrate that non-conserved residues within this region, such T606 and K370 in TMEM16F, are important for the inhibitory effects of 1PBC and/or niclosamide (Fig. 3). In fact, comparison between the two pockets reveals that the TMEM16A binding pocket consists almost exclusively of hydrophobic residues, whereas the equivalent site in TMEM16F includes several charged and OH-containing side chains (Fig. 5B). Niclosamide is a highly hydrophobic molecule that presents extremely poor solubility in aqueous solutions (37). Thus, the identification of non-conserved hydrophilic residues within the drug binding pocket in TMEM16F opens the door for the development of niclosamide analogs with better pharmacological properties that exclusively target TMEM16F for the treatment of severe COVID-19. Taken together, our work establishes a much-needed structural framework for designing more potent and more specific antagonists against individual members of the TMEM16 family with critical implications for the treatment of asthma, cancer and COVID-19.

## Supplementary Material

1

## References

[1] Falzone M. E., Malvezzi M., Lee B. C., Accardi A., Known structures and unknown mechanisms of TMEM16 scramblases and channels. J Gen Physiol 150, 933–947 (2018). 29915161 10.1085/jgp.201711957 PMC6028493

[2] Kalienkova V., Clerico Mosina V., Paulino C., The Groovy TMEM16 Family: Molecular Mechanisms of Lipid Scrambling and Ion Conduction. J Mol Biol 433, 166941 (2021). 33741412 10.1016/j.jmb.2021.166941

[3] Caputo A. , TMEM16A, a membrane protein associated with calcium-dependent chloride channel activity. Science 322, 590–594 (2008). 18772398 10.1126/science.1163518

[4] Yang Y. D. , TMEM16A confers receptor-activated calcium-dependent chloride conductance. Nature 455, 1210–1215 (2008). 18724360 10.1038/nature07313

[5] Schroeder B. C., Cheng T., Jan Y. N., Jan L. Y., Expression cloning of TMEM16A as a calcium-activated chloride channel subunit. Cell 134, 1019–1029 (2008). 18805094 10.1016/j.cell.2008.09.003 PMC2651354

[6] Yang H. , TMEM16F forms a Ca^2+^-activated cation channel required for lipid scrambling in platelets during blood coagulation. Cell 151, 111–122 (2012). 23021219 10.1016/j.cell.2012.07.036 PMC3582364

[7] Han T. W. , Chemically induced vesiculation as a platform for studying TMEM16F activity. Proc Natl Acad Sci U S A 116, 1309–1318 (2019). 30622179 10.1073/pnas.1817498116 PMC6347726

[8] Feng S. , Cryo-EM studies of TMEM16F calcium-activated ion channel suggest features important for lipid scrambling. Cell Rep 28, 567–579 e564 (2019). 31291589 10.1016/j.celrep.2019.06.023 PMC6684876

[9] Ye W., Han T. W., He M., Jan Y. N., Jan L. Y., Dynamic change of electrostatic field in TMEM16F permeation pathway shifts its ion selectivity. Elife 8, (2019). 10.7554/eLife.45187 PMC6690719 31318330

[10] Alvadia C. , Cryo-EM structures and functional characterization of the murine lipid scramblase TMEM16 F. Elife 8, (2019). 10.7554/eLife.44365 PMC6414204 30785399

[11] Watanabe R., Sakuragi T., Noji H., Nagata S., Single-molecule analysis of phospholipid scrambling by TMEM16F. Proc Natl Acad Sci U S A 115, 3066–3071 (2018). 29507235 10.1073/pnas.1717956115 PMC5866571

[12] Chen W. , The Prognostic Value and Mechanisms of TMEM16 A in Human Cancer. Front Mol Biosci 8, 542156 (2021). 33681289 10.3389/fmolb.2021.542156 PMC7930745

[13] Braga L. , Drugs that inhibit TMEM16 proteins block SARS-CoV-2 spike-induced syncytia. Nature 594, 88–93 (2021). 33827113 10.1038/s41586-021-03491-6 PMC7611055

[14] Danahay H. L. , TMEM16A Potentiation: A Novel Therapeutic Approach for the Treatment of Cystic Fibrosis. Am J Respir Crit Care Med 201, 946–954 (2020). 31898911 10.1164/rccm.201908-1641OC PMC7159426

[15] Cabrita I., Benedetto R., Schreiber R., Kunzelmann K., Niclosamide repurposed for the treatment of inflammatory airway disease. JCI Insight 4, (2019). 10.1172/jci.insight.128414 PMC6693830 31391337

[16] Miner K. , Drug Repurposing: The Anthelmintics Niclosamide and Nitazoxanide Are Potent TMEM16A Antagonists That Fully Bronchodilate Airways. Front Pharmacol 10, 51 (2019). 30837866 10.3389/fphar.2019.00051 PMC6382696

[17] Fujii T., Sakata A., Nishimura S., Eto K., Nagata S., TMEM16F is required for phosphatidylserine exposure and microparticle release in activated mouse platelets. Proc Natl Acad Sci U S A 112, 12800–12805 (2015). 26417084 10.1073/pnas.1516594112 PMC4611630

[18] Wolf P, The nature and significance of platelet products in human plasma. Br J Haematol 13, 269–288 (1967). 6025241 10.1111/j.1365-2141.1967.tb08741.x

[19] Zwaal R. F., Comfurius P., Bevers E. M., Scott syndrome, a bleeding disorder caused by defective scrambling of membrane phospholipids. Biochim Biophys Acta 1636, 119–128 (2004). 15164759 10.1016/j.bbalip.2003.07.003

[20] Suzuki J., Umeda M., Sims P. J., Nagata S., Calcium-dependent phospholipid scrambling by TMEM16F. Nature 468, 834–838 (2010). 21107324 10.1038/nature09583

[21] Gyorgy B. , Membrane vesicles, current state-of-the-art: emerging role of extracellular vesicles. Cell Mol Life Sci 68, 2667–2688 (2011). 21560073 10.1007/s00018-011-0689-3 PMC3142546

[22] Raposo G., Stoorvogel W., Extracellular vesicles: exosomes, microvesicles, and friends. J Cell Biol 200, 373–383 (2013). 23420871 10.1083/jcb.201211138 PMC3575529

[23] Sims P. J., Wiedmer T., Esmon C. T., Weiss H. J., Shattil S. J., Assembly of the platelet prothrombinase complex is linked to vesiculation of the platelet plasma membrane. Studies in Scott syndrome: an isolated defect in platelet procoagulant activity. J Biol Chem 264, 17049–17057 (1989). 2793843

[24] Whitlock J. M., Hartzell H. C., Anoctamins/TMEM16 Proteins: Chloride Channels Flirting with Lipids and Extracellular Vesicles. Annu Rev Physiol 79, 119–143 (2017). 27860832 10.1146/annurev-physiol-022516-034031 PMC5556385

[25] Wu N. , Critical Role of Lipid Scramblase TMEM16F in Phosphatidylserine Exposure and Repair of Plasma Membrane after Pore Formation. Cell Rep 30, 1129–1140 e1125 (2020). 31995754 10.1016/j.celrep.2019.12.066 PMC7104872

[26] Abdulamir A. S. , A randomised controlled trial of effectiveness and safety of Niclosamide as add on therapy to the standard of care measures in COVID-19 management. Ann Med Surg (Lond) 69, 102779 (2021). 34512959 10.1016/j.amsu.2021.102779 PMC8416702

[27] Dang S. , Cryo-EM structures of the TMEM16A calcium-activated chloride channel. Nature 552, 426–429 (2017). 29236684 10.1038/nature25024 PMC5750132

[28] Paulino C., Kalienkova V., Lam A. K. M., Neldner Y., Dutzler R., Activation mechanism of the calcium-activated chloride channel TMEM16A revealed by cryo-EM. Nature 552, 421–425 (2017). 29236691 10.1038/nature24652

[29] Arreola J., Hartzell H. C., Wasted TMEM16A channels are rescued by phosphatidylinositol 4,5-bisphosphate. Cell Calcium 84, 102103 (2019). 31683182 10.1016/j.ceca.2019.102103 PMC6913893

[30] Le S. C., Jia Z., Chen J., Yang H., Molecular basis of PIP2-dependent regulation of the Ca(2+)-activated chloride channel TMEM16A. Nat Commun 10, 3769 (2019). 31434906 10.1038/s41467-019-11784-8 PMC6704070

[31] Ye W. , Phosphatidylinositol-(4, 5)-bisphosphate regulates calcium gating of small-conductance cation channel TMEM16F. Proc Natl Acad Sci U S A 115, E1667–E1674 (2018). 29382763 10.1073/pnas.1718728115 PMC5816197

[32] Gao Y., Cao E., Julius D., Cheng Y., TRPV1 structures in nanodiscs reveal mechanisms of ligand and lipid action. Nature 534, 347–351 (2016). 27281200 10.1038/nature17964 PMC4911334

[33] Schuler M. A., Denisov I. G., Sligar S. G., Nanodiscs as a new tool to examine lipid-protein interactions. Methods Mol Biol 974, 415–433 (2013). 23404286 10.1007/978-1-62703-275-9_18 PMC4201044

[34] Bushell S. R. , The structural basis of lipid scrambling and inactivation in the endoplasmic reticulum scramblase TMEM16K. Nat Commun 10, 3956 (2019). 31477691 10.1038/s41467-019-11753-1 PMC6718402

[35] Le S. C., Yang H., An Additional Ca(2+) Binding Site Allosterically Controls TMEM16A Activation. Cell Rep 33, 108570 (2020). 33378669 10.1016/j.celrep.2020.108570 PMC7786149

[36] Biton B. , The antipsychotic drug loxapine is an opener of the sodium-activated potassium channel slack (Slo2.2). J Pharmacol Exp Ther 340, 706–715 (2012). 22171093 10.1124/jpet.111.184622

[37] Chen W., Mook R. A. Jr., Premont R. T., Wang J., Niclosamide: Beyond an antihelminthic drug. Cell Signal 41, 89–96 (2018). 28389414 10.1016/j.cellsig.2017.04.001 PMC5628105

[38] Mastronarde D. N., Automated electron microscope tomography using robust prediction of specimen movements. J Struct Biol 152, 36–51 (2005). 16182563 10.1016/j.jsb.2005.07.007

[39] Tan Y. Z. , Addressing preferred specimen orientation in single-particle cryo-EM through tilting. Nat Methods 14, 793–796 (2017). 28671674 10.1038/nmeth.4347 PMC5533649

[40] Zheng S. Q. , MotionCor2: anisotropic correction of beam-induced motion for improved cryo-electron microscopy. Nat Methods 14, 331 −332 (2017). 28250466 10.1038/nmeth.4193 PMC5494038

[41] Punjani A., Rubinstein J. L., Fleet D. J., Brubaker M. A., cryoSPARC: algorithms for rapid unsupervised cryo-EM structure determination. Nat Methods 14, 290–296 (2017). 28165473 10.1038/nmeth.4169

[42] P Emsley K. Cowtan, Coot: model-building tools for molecular graphics. Acta Crystallogr D Biol Crystallogr 60, 2126–2132 (2004). 15572765 10.1107/S0907444904019158

[43] Afonine P. V. , Towards automated crystallographic structure refinement with phenix.refine. Acta Crystallogr D Biol Crystallogr 68, 352–367 (2012). 22505256 10.1107/S0907444912001308 PMC3322595

[44] Friesner R. A. , Glide: a new approach for rapid, accurate docking and scoring. 1. Method and assessment of docking accuracy. J Med Chem 47, 1739–1749 (2004). 15027865 10.1021/jm0306430

[45] Harder et a E.. OPLS3: A Force Field Providing Broad Coverage of Drug-like Small Molecules and Proteins. J Chem Theory Comput 12, 281–296 (2016). 26584231 10.1021/acs.jctc.5b00864

[46] Shelley et a J. C.. Epik: a software program for pK(a) prediction and protonation state generation for drug-like molecules. J Comput Aided Mol Des 21, 681 −691 (2007). 17899391 10.1007/s10822-007-9133-z

[47] Friesner R. A. , Extra precision glide: docking and scoring incorporating a model of hydrophobic enclosure for protein-ligand complexes. J Med Chem 49, 6177–6196 (2006). 17034125 10.1021/jm051256o

