# Supplementary material for: Identification of a conserved drug binding pocket in TMEM16 proteins
Source: Res Sq. 2022 Feb 10:rs.3.rs-1296933. Preprint. [Version 1] doi: 10.21203/rs.3.rs-1296933/v1 (PMC8845511; doi:10.21203/rs.3.rs-1296933/v1)
Supplement: 1 [file 294acf08c2b90d8de7f0d335.docx]

**Supplementary materials**

Materials and Methods

*Protein expression and purification*

TMEM16F was expressed and prepared as previously described (*8*) with some modifications. Briefly, the protein was expressed in HEK293s cells, extracted and purified in the presence of DDM/CHS and reconstituted in MSP2N2-SoyPC nanodiscs at a 1:4:100 molar ratio of TMEM16 monomer:MSP:lipid. PIP_2_ was added to the sample after nanodisc reconstitution at a 4:1 molar ratio of PIP_2_:TMEM16 monomer and incubated for 15 min before FPLC. 1-oxo-3-(trifluoromethyl)-1,5-dihydropyrido[1,2-a]benzimidazole-4-carbonitrile (1PBC) was purchased from VITAS-M laboratory (Champaign, IL, USA) and niclosamide was purchased from Sigma Aldrich (St. Louis, MO, USA). 50 and 25 mM stocks of 1PBC and niclosamide were freshly prepared in DMSO and EtOH, respectively, and added to the sample to a final concentration of 100 and 50 µM, respectively. The inhibitors and 4mM CaCl_2_ were added to the sample immediately prior to grid preparation.

*Sample preparation for electron microscopy*

For cryo-EM structure determination, 3.5 µl of the sample at approx. 0.7 mg/ml were applied to 300 mesh UltrAuFoil Holey Gold Films R1.2/1.3 (Quantifoil) that had been previously plasma cleaned. The grids were loaded into a Vitrobot (ThermoFisher) with an environment chamber at a temperature of 4°C and 100% humidity, blotted for 4 seconds at 0 blotting force with Whatman No.4 filter paper and plunged into a liquid ethane slurry.

*Electron microscopy data acquisition*

Cryo-EM data were collected at Janelia HHMI Cryo-EM facility on a ThermoFisher Krios transmission electron microscope (TEM) operating at 300 keV with a Quantum energy filter (Gatan) set to a slit width of 20 eV. Dose-fractionated movies were collected using a Gatan K3 Summit direct electron detector operating in CDS super resolution mode. Collection dose rate was 9 e^-^/pixel/second and the total cumulative dose was 66 e^-^/Å^2^ over 120 frames. Micrographs were collected with a 3x3 image shift collection strategy at 105000x magnification (0.42 Å/pixel at the specimen level in super resolution mode), with a nominal defocus range of -0.8 to -2.2 μm using semi-automated scripts in SerialEM (*38*). All samples presented severe preferred orientation. To address this issue, images were collected at 0º, 30º, 35º and 45º degrees tilt (*39*) as specified in table S1.

*Image processing*

For each dataset, micrograph frames were aligned using MotionCorr2 (*40*) and cryoSPARC 3.0 (*41*) was used for all initial processing: CTF was estimated with CTF Patch and only micrographs with resolution estimations higher than 3.5 Å with confidence values above 97% were further processed. Particles were picked using BlobPicker and extracted with an unbinned box size of 256 pixels. 2D classification was used to eliminate obvious mispicks and selected particles were reconstructed using non-uniform refinement using our previous cryoEM reconstruction of TMEM16F (EMD-20246), respectively, low pass filtered to 30 Å as an initial model. Tilted and untilted datasets were merged and subjected to heterogenous refinement using 3 classes. Of note, as initial models for the heterogenous refinement, we inputted 2 volumes representing of our previous reconstructions, whereas the initial model for class 3 was a volume obtained from a failed ab initio reconstruction. The latter captured empty nanodiscs and damaged particles, allowing us to classify those out. Successive rounds of this processing pipeline rendered high resolution reconstructions that were further processed using local refinement in cryoSPARC. For analysis of the drug binding site in TMEM16F, we performed focused classification without alignment in Relion 3.1. with a Tau value of 20 using a mask around the ligand-binding area. The outputs were subjected to a manual refinement in cisTEM.

*Atomic model building and refinement*

Previous structures of TMEM16F (PDB: 6P48) were used as a starting model. The structure was adjusted and novel areas were built de novo using the COOT software package (*42*), and further refined using ISOLDE and real-space refinement from the PHENIX package (*43*). Chimera X was used for visualization and figures.

#### *Lipid scrambling assay*

#### Lipid scrambling was assayed as previously reported (*7, 8*) with some modifications. Briefly, Stable HEK293 cell lines expressing wild-type or mutant mTMEM16F were plated on glass bottom dishes in Opti-MEM medium overnight prior to imaging. Cells were washed twice in HEPES-buffered modified Tyrode’s buffer (10 mM HEPES pH7.5, 150 mM NaCl, 10 mM glucose, 2 mM CaCl_2_) and incubated in the same buffer containing 25 mM paraformaldehyde (PFA)/and 1:100 pSIVA (BioRad, Hercules, CA) for 5 min. Image acquisition began once dithiothreitol (DTT) was added to a final concentration of 2 mM and terminated after 1 hour. Image analysis was carried out on each individual cell using the Nikon Elements Software (*7*). To calculate the values for time of onset, we fit the pSIVA imaging curve with the Weibull growth model using Graph Pad Prism 9: Y=YM−(YM−Y0)∗exp(−1∗((k∗x)g). The time of onset is the maximum of the second derivative.

#### *Ca^2+^ rise measurement*

#### Cultured cells were incubated with 1 μM of the calcium reporter dye Fluo-8 AM (AAT Bioquest) for 15 min, washed twice in Dulbecco’s [phosphate buffered saline](https://www.sciencedirect.com/topics/neuroscience/phosphate-buffered-saline) (DPBS), and treated with 25 mM PFA/2 mM DTT in HEPES-buffered modified Tyrode’s buffer (10 mM HEPES pH7.5, 150 mM NaCl, 10 mM glucose, 2 mM CaCl_2_) for live [cell imaging](https://www.sciencedirect.com/topics/biochemistry-genetics-and-molecular-biology/cellular-imaging) on a Nikon-TE2000 inverted microscope (Nikon Instruments, Melville, NY, USA) equipped with a [thermostat](https://www.sciencedirect.com/topics/agricultural-and-biological-sciences/thermostats) chamber. Images for both brightfield and Fluo-8 were acquired in parallel once every minute starting 5 min post-treatment for 50 min for a total of 55 min treatment time. To calculate the values for time of onset, we fit the F/F0 curve with the Weibull growth model using Graph Pad Prism 9: Y=YM−(YM−Y0)∗exp(−1∗((k∗x)g). The time of onset is the maximum of the second derivative.

*Electrophysiology*

Cells were seeded in a 12-well plate for whole-cell patch clamp recordings and transfected the following day using the FuGENE^®^ 6 Transfection Reagent (Promega, U.S.A.) following manufacturer’s instructions. All experiments were performed 20-30 h after transfection. The cells were transferred onto a small chamber on the stage of an inverted microscope (TE2000, Nikon, Japan) and attached to coverslip in the small chamber for 10 min prior to the patch recording. Experiments were performed at room temperature (22-24˚C). The recording chamber was continuously perfused at a flow rate of 1-2 ml/min. Borosilicate patch pipettes (Sutter Instrument, Novato, CA, USA) of 3 - 5 MΩ resistance were pulled from a Sutter P-1000 puller and used to obtain giga-ohm seals for whole cell patch configuration. The currents were recorded using an Axopatch 200B patch-clamp amplifier (Molecular Devices, Foster City, CA, USA). pClamp software v11.2 and Digidata 1440B (Molecular Devices) were used for data acquisition and application of the pulses. Low-pass Bessel filter with 5kHz cut-off frequency was selected for recordings. Pipette capacitance cancellation up to 90% was achieved in cell-attached modes of every recording. The data were analyzed using pCLAMP software v11.2, OriginPro 8 (OriginLab, Northampton, MA, USA). The bath solution was perfused constantly with buffer containing 10 mM HEPES pH 7.2, 145 mM NaCl, 2 mM MgCl_2_, 1 mM CaCl_2_ and 10 mM Glucose. The patch pipette solution contained 141.24 or 140.06 mM CsCl, 10 mM HEPES, 5 mM EGTA, 1 mM MgCl_2_, 3.76 or 4.94 mM CaCl_2_ ([Ca^2+^]_i_ = 0.5 or 12 μM, respectively), pH 7.2 with CsOH. The free calcium concentrations were calculated using the MaxChelator WEBMAXC Standard program. 1PBC and niclosamide were freshly dissolved in DMSO and EtOH, respectively, and each stock was diluted to the final concentration in bath solution.

*Docking*

We used Glide (Schrödinger, Inc.) (*44*) in the docking study. The protein was prepared with a default protocol from Protein Preparation Wizard and OPLS3 force field (*45*). We applied Epik (*46*) method in the ligand preparation for both 1PBC and niclosamide with the range of pH from 6.4 to 8.4. Standard precision (SP) and extra precision (XP) (*47*) docking protocols were used to generate binding poses for both compounds within a 30x30x30 Å box that encompasses most of the TM region. Top ranking positions within the box were determined based on favorable hydrogen binding interactions and hydrophobic forces.

**Table S1. Cryo-EM data collection and refinement statistics**

| Sample | TMEM16F  control | | | TMEM16F  Niclosamide | TMEM16F  1PBC |
| --- | --- | --- | --- | --- | --- |
| State | State B | Class 1 | Class 2 |  |  |
| EMDB | xxx | xxx | xxx | xxxx | xxxx |
| PDB | xxx | xxx | xxx | xxx | xxx |
| **Data collection and processing** |  | | | | |
| Microscope/ Detector | Titan Krios/Gatan K3 with Gatan Bioquantum energy filter | | | | |
| Imaging software and collection | SerialEM, 3x3 image shift | | | | |
| Magnification | 105,000 | | | | |
| Voltage (kV) | 300 | | | | |
| Electron exposure (e^-^/Å^2^) | 66 | | | | |
| Dose rate (e^-^/pix/sec) | 8 | | | | |
| Frame exposure (e^-^/Å^2^) | 0.55 | | | | |
| Defocus range (μm) | (-0.8) - (-2.2) | | | | |
| Pixel size (Å) | 0.839 | | | | |
| Micrographs | 2526 (0º tilt)  6348 (30º tilt) | | | 4404 (0º tilt)  5359 (35º tilt) 5474 (45º tilt) | 2160 (0º tilt) 3062 (35º tilt) |
|  |  |  |  |  |  |
| **Reconstruction** |  |  |  |  |  |
| Picked particles |  |  |  |  |  |
| (Blob picker in cryosparc) |  |  |  |  |  |
| Particles in final refinement | 322,295 | 426,618 | 539,866 | 250,553 | 203,646 |
|  |  |  |  |  |  |
| Symmetry imposed | C1 | C1 | C1 | C1 | C1 |
| Guinier plot B factor (1/Å^2^) | 152.1 | 158.8 | 158.9 |  | 135.8 |
| Map resolution, global FSC (Å) |  |  |  |  |  |
| FSC 0.143, unmasked/masked | 3.7/3.2 | 3.7/3.2 | 3.5/3.1 | 3.3/3.1 | 3.6/2.9 |
|  |  |  |  |  |  |
| **Refinement** |  |  |  |  |  |
| Initial model used, PDB code | 6P48 | | | | |
| Model resolution (Å) |  |  |  |  |  |
| FSC 0.5 unmasked/masked | 3.5/3.4 | 3.4/3.3 | 3.7/3.5 | 3.4/3.3 | 3.6/3.5 |
| Model composition |  |  |  |  |  |
| Non-hydrogen atoms | 23599 | 23599 | 23599 | 23793 | 23624 |
| Protein residues | 1448 | 1448 | 1448 | 1448 | 1448 |
| Glycans (NAG) | 6 | 6 | 6 | 12 | 6 |
| *B* factors (Å^2^) |  |  |  |  |  |
| Protein | 193.10 | 79.69 | 76.03 | 143.92 | 88.38 |
| Ligand | 197.07 | 79.85 | 80.12 | 152.33 | 80.69 |
| R.m.s. deviations |  |  |  |  |  |
| Bond lengths (Å) | 0.004 | 0.003 | 0.004 | 0.004 | 0.003 |
| Bond angles (º) | 0.712 | 0.547 | 0.589 | 0.776 | 0.572 |
| Validation |  |  |  |  |  |
| MolProbity score | 1.47 | 1.72 | 1.85 | 1.41 | 1.80 |
| Clashscore | 4.07 | 6.97 | 5.65 | 3.95 | 6.13 |
| Poor rotamers (%) | 0 | 0 | 0 | 0 | 0 |
| EMRinger score | 2.08 | 3.00 | 2.41 | 3.08 | 1.92 |
| CaBLAM score | 2.35 | 1.99 | 1.99 | 1.57 | 1.85 |
| Ramachandran plot |  |  |  |  |  |
| Favored (%) | 96.00 | 95.79 | 95.37 | 96.49 | 95.23 |
| Allowed (%) | 3.93 | 4.21 | 4.49 | 3.30 | 4.63 |
| Disallowed (%) | 0.07 | 0.00 | 0.14 | 0.21 | 0.14 |

**
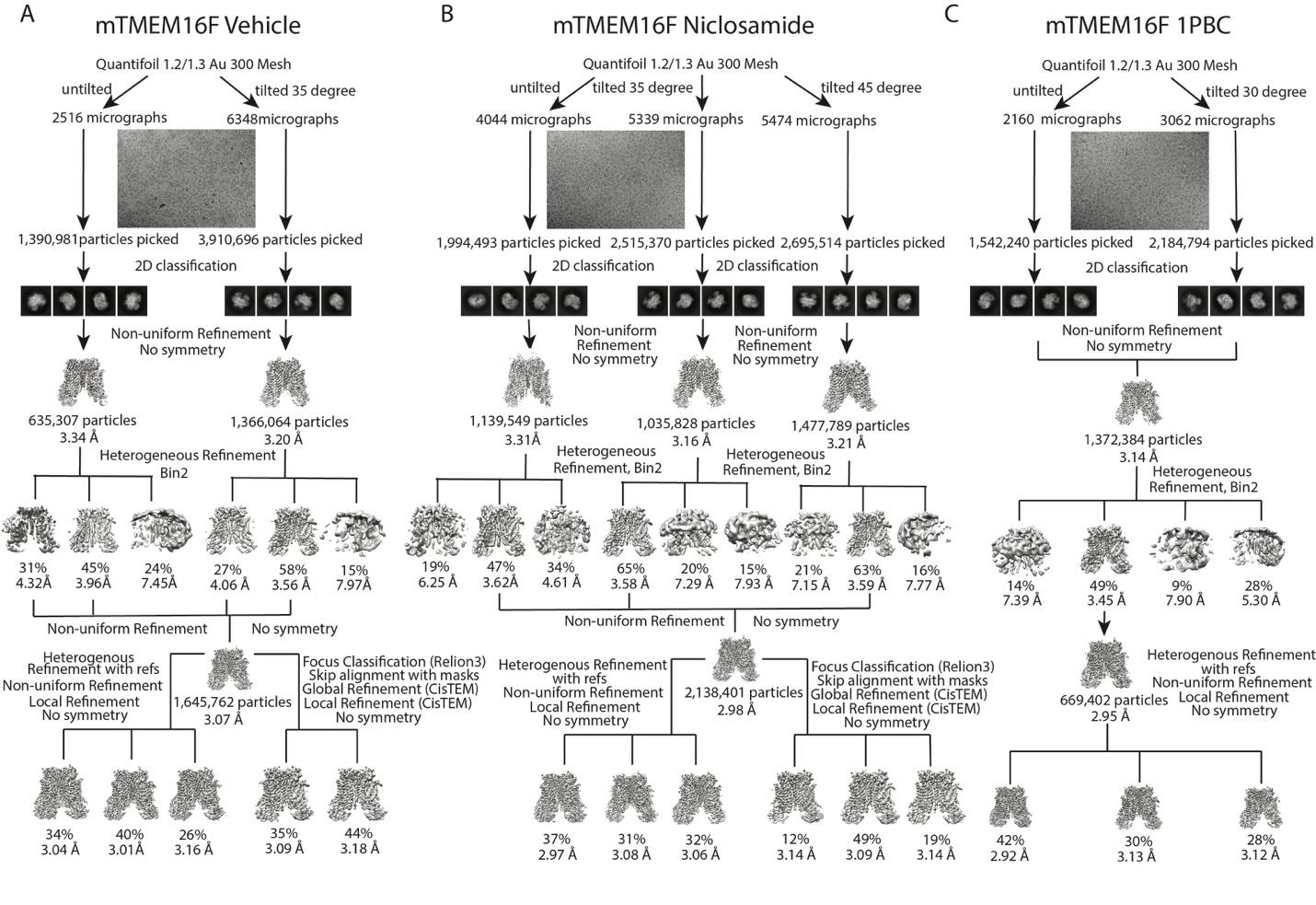
**

**Figure S1. Cryo-EM workflow.** Representative micrographs and processing pipeline for TMEM16F purified in the absence of inhibitors (**A**) or in the presence of (**B**) niclosamide or (**C**) 1PBC. Briefly, micrographs were collected at different tilting angles and CTF correction was performed using CTF Patch in cryoSPARC. Particles were picked using Blobpicker, followed by 2D classification and Non-uniform refinement. Several rounds of consecutive Heterogenous refinement and non-uniform refinement rendered 3 high resolution classes (States A, B and C). Particles were subjected to focused classification without alignment in Relion 3.1 using a mask around the TM1-TM6 groove and the resulting classes underwent a final round of C1 refinement in cisTEM.

**
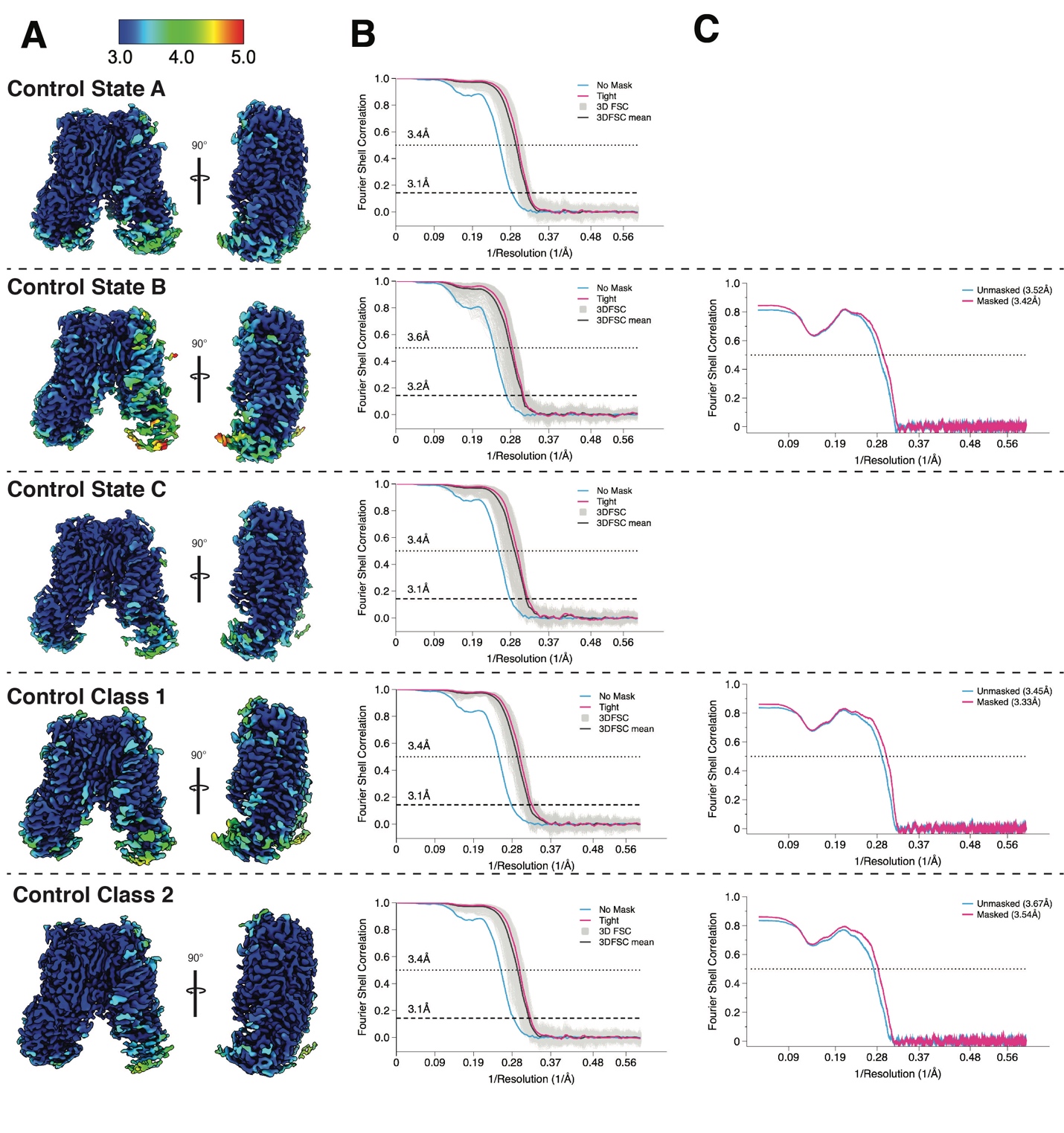
**

**Figure S2. Validation of cryo-EM maps and models of unliganded TMEM16F.** (**A**) Local resolution estimates of TMEM16F reconstructions as reported by cryoSPARC with all maps colored on the same scale, as indicated. (**B**) Golden Standard Fourier Shell Correlation (GSFSC) and 3DFSC plots for cryo-EM maps. Resolution values at FSC=0.143 and 0.5 are noted. (**C)** Model-map correlation calculated in Phenix with resolution values at FSC= 0.5 in parenthesis.


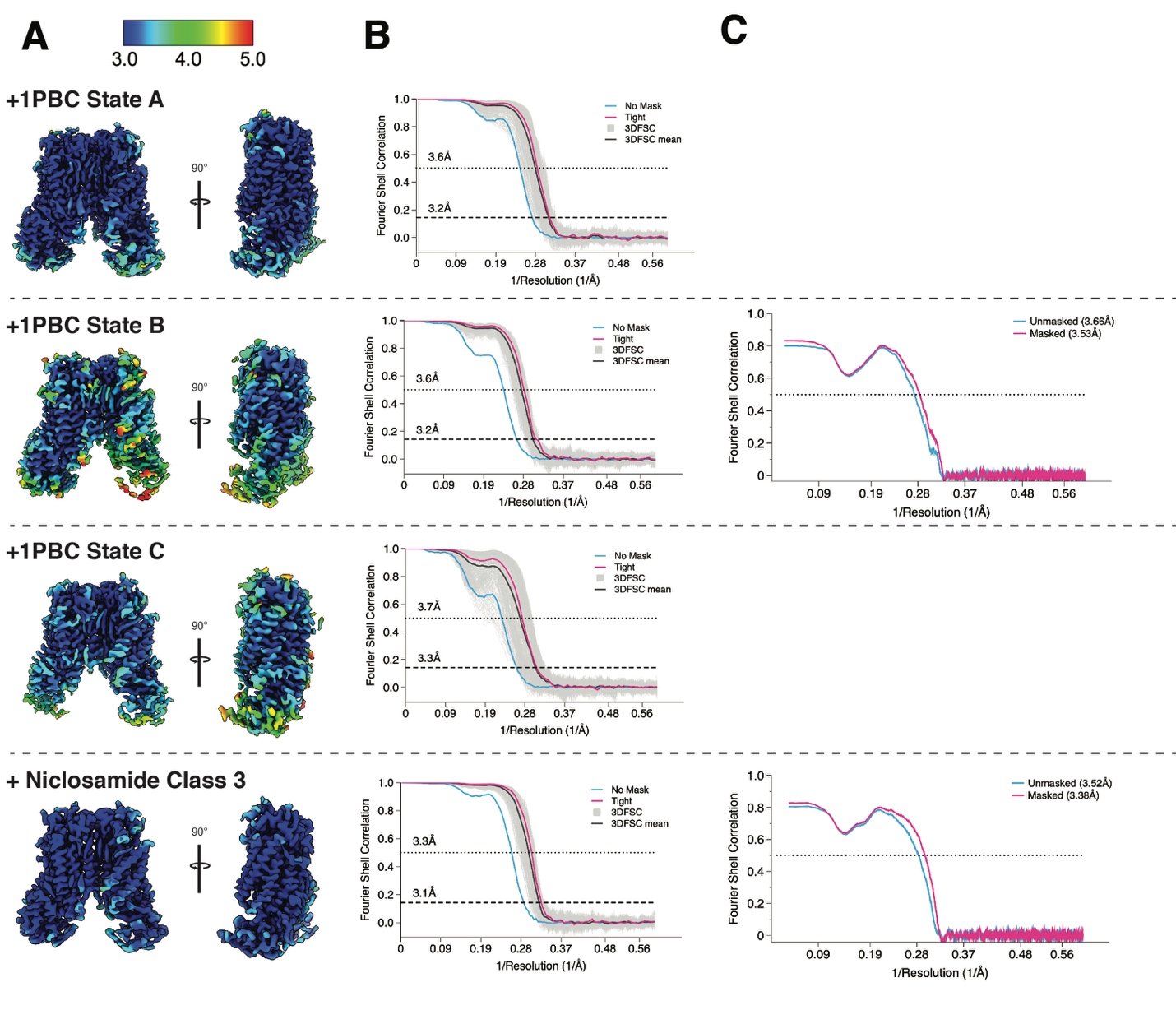


**Figure S3. Validation of cryo-EM maps and models of TMEM16F in the presence of inhibitors.** (**A**) Local resolution estimates of TMEM16F reconstructions as reported by cryoSPARC with all maps colored on the same scale, as indicated. (**B**) Golden Standard Fourier Shell Correlation (GSFSC) and 3DFSC plots for cryo-EM maps. Resolution values at FSC=0.143 and 0.5 are noted. (**C)** Model-map correlation calculated in Phenix with resolution values at FSC= 0.5 in parenthesis.

**
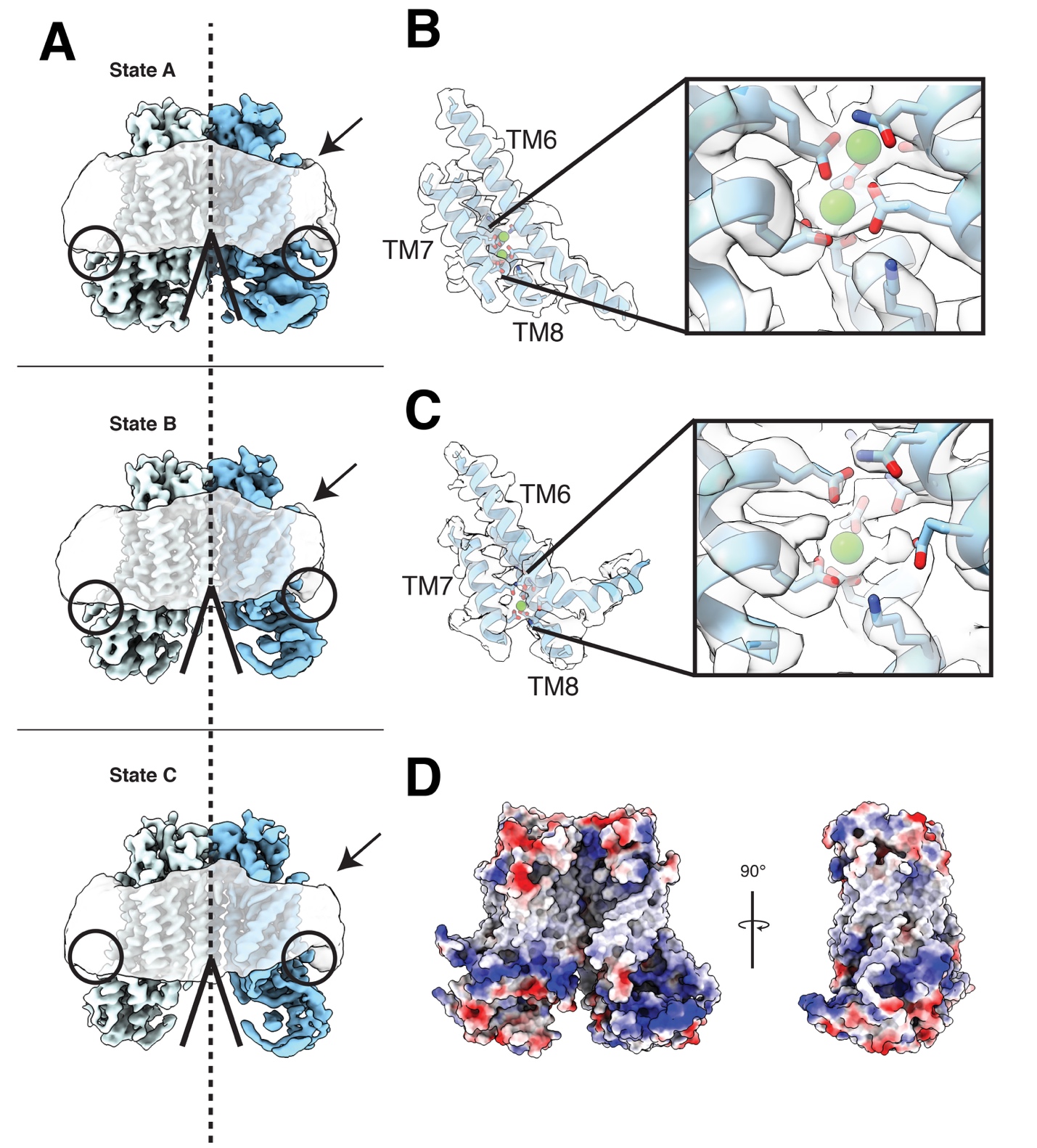
**

**Figure S4. Identification of three distinct states in drug-free TMEM16F. (A)** Gaussian filtered density of the nanodisc and unsharpened density of the protein dimer. Unsharpened cryo-EM density and atomic model for (**B**) the extended TM6 and (**C**) the kinked TM6. Right, zoom into the Ca^2+^ binding site with the sharpened cryo-EM density shown in semitransparent and the residues depicted as sticks colored by heteroatom. (**D)** Electrostatic surface of the asymmetric TMEM16F dimer, where white represents hydrophobic areas and blue and red corresponds to positively and negatively charged regions, respectively.

**
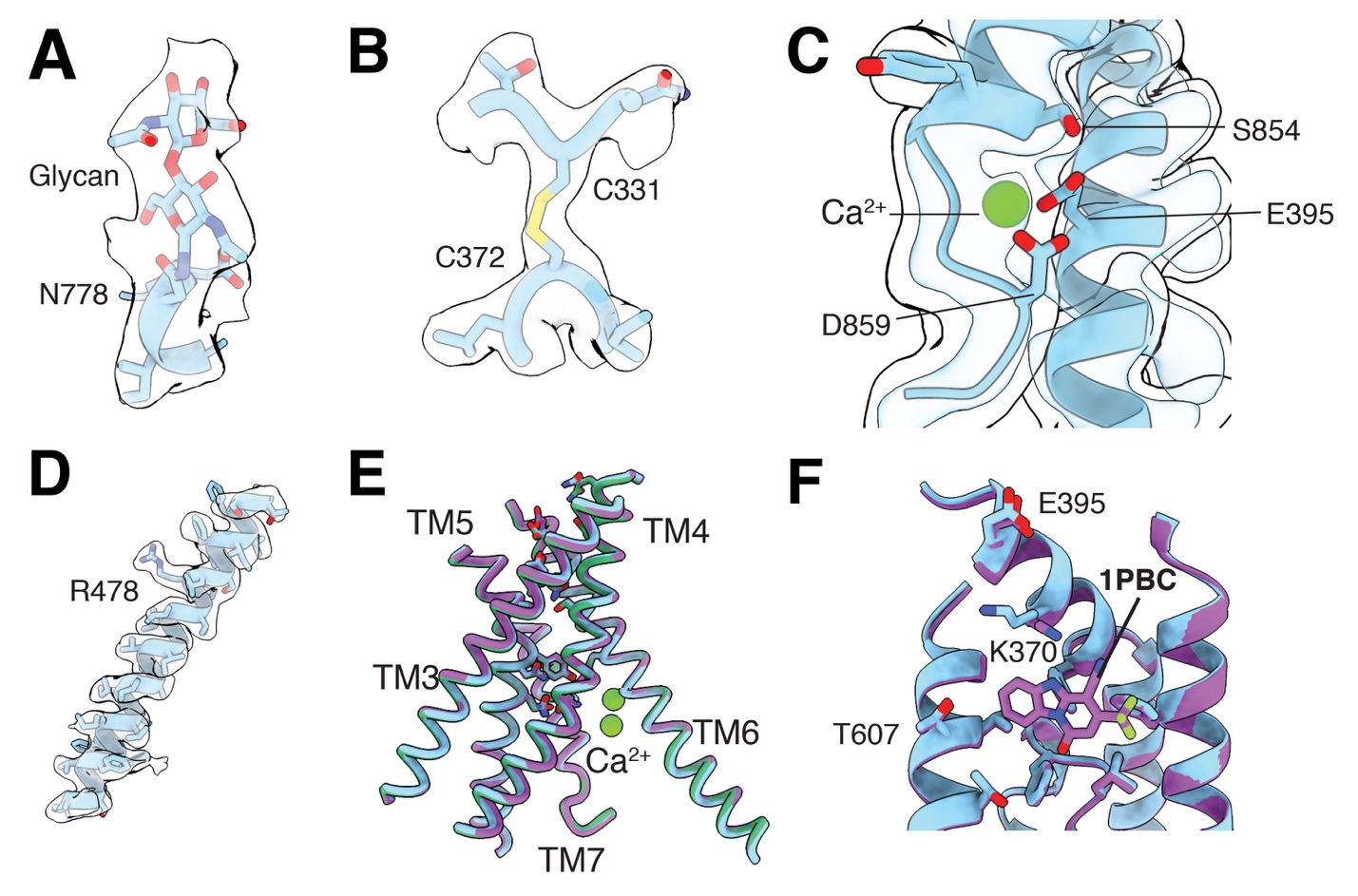
Figure S5.** **Structural details of TMEM16F.** Atomic model for (**A**) N-glycosylation at N778, (**B**) disulphide bond between residues C331 and C373, (**C**) third Ca^2+^ binding site at the dimer interface and (**D**) TM4. In all cases, the cryo-EM density is shown in semitransparent and the residues are colored by heteroatom. Overlay of (**E**) the ion conduction channel and (**F**) the drug binding site in unliganded (blue), niclosamide- (green) and 1PBC-bound (purple) structures of TMEM16F.

**
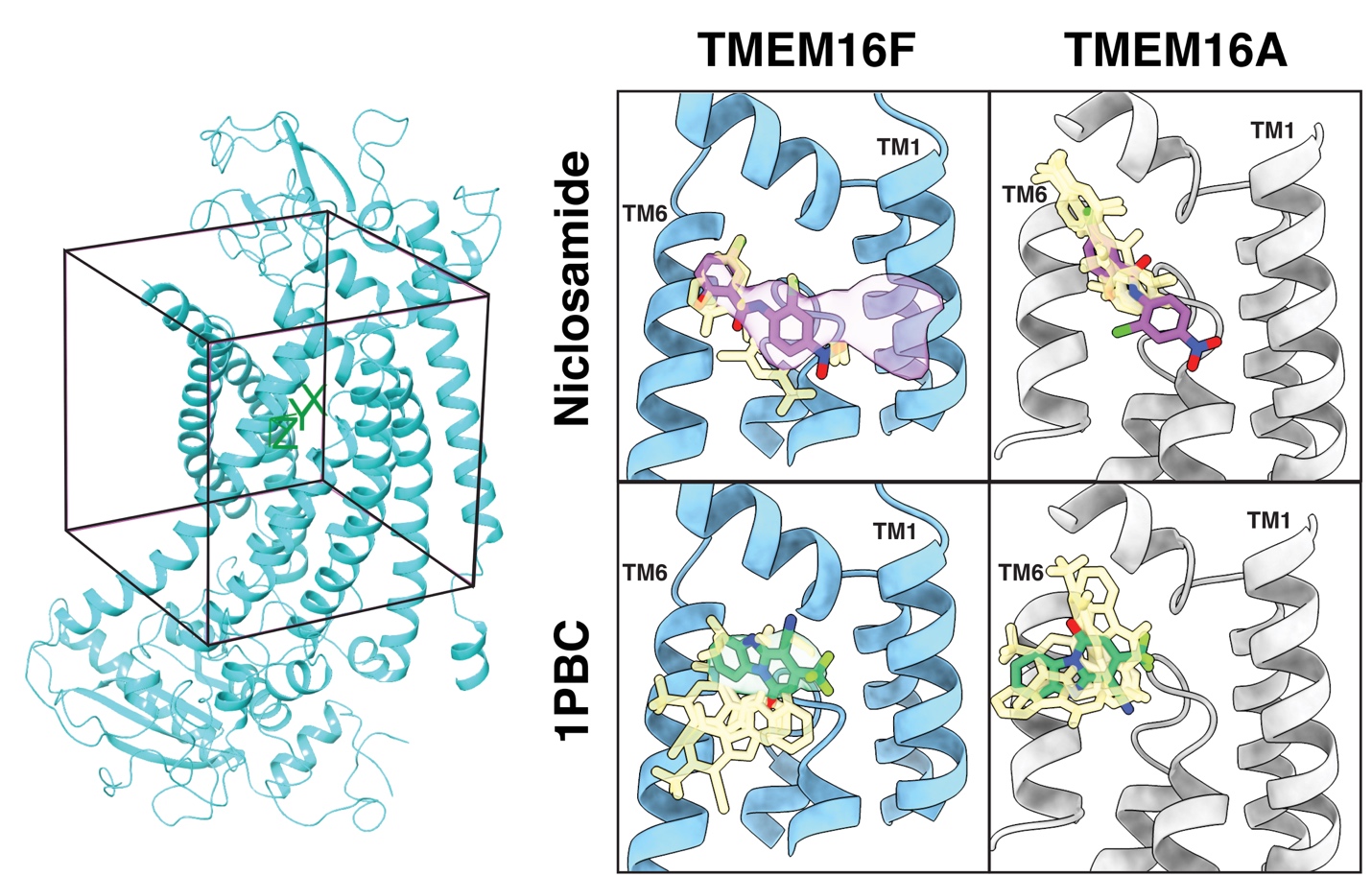
**

**Figure S6.** **Computational docking of niclosamide in 1PBC in TMEM16s.** The Glide software was used to computationally dock niclosamide and 1PBC into the boxed area in the atomic model of TMEM16F (left) and TMEM16A (5OYB, right). The top 5 ranking poses of niclosamide (top) and 1PBC (bottom) are shown in yellow with the best fitting position highlighted in purple and green, respectively.

**
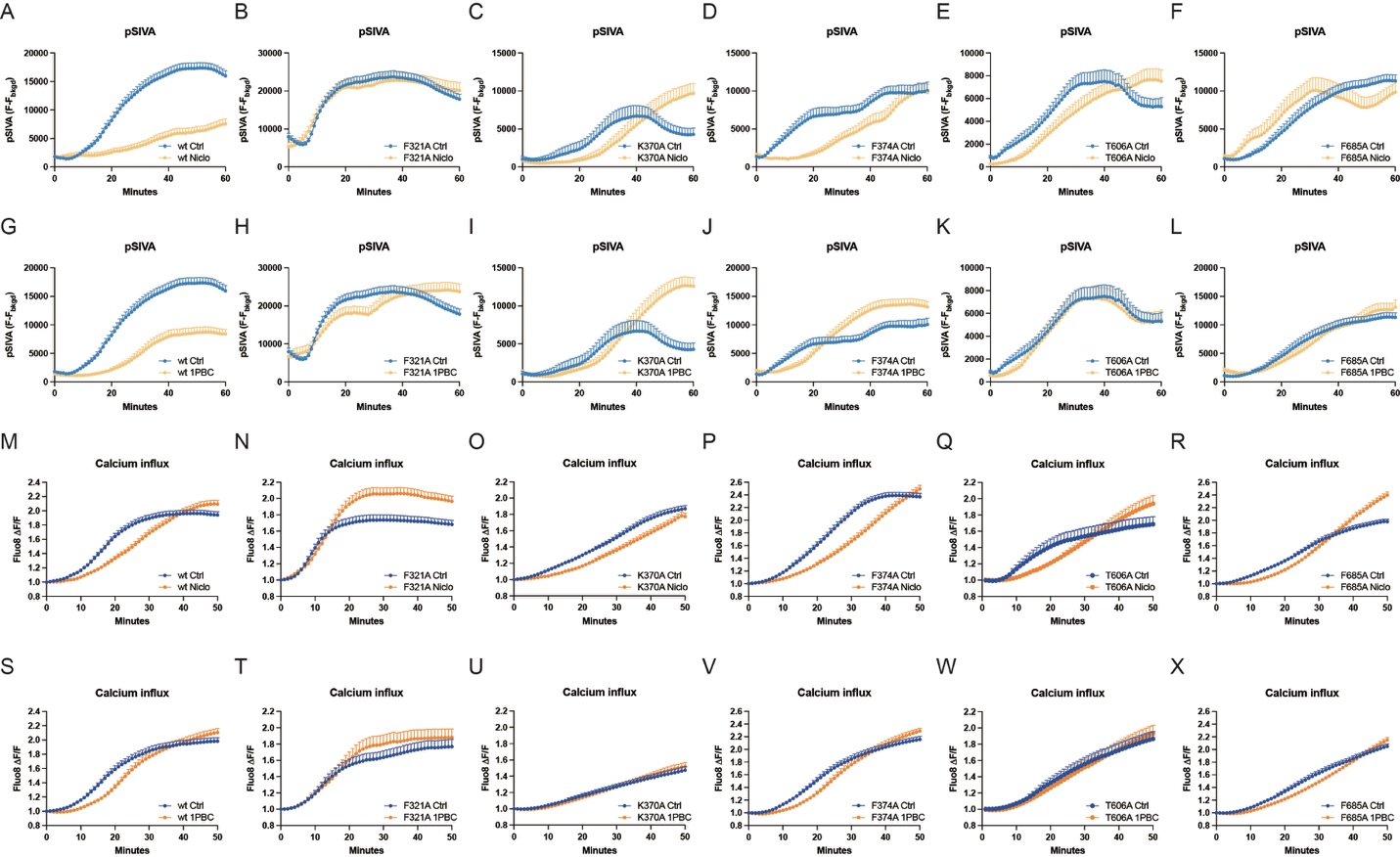
**

**Figure S7. Functional validation for TMEM16F**

Representative curves of live imaging of TMEM16F-dependent PS exposure (**A** - **L**) and Ca^2+^ influx (**M** - **X**). Data are represented as mean ± SEM.
